# Olanzapine-Associated Rhabdomyolysis: A Case Report

**DOI:** 10.7759/cureus.12568

**Published:** 2021-01-08

**Authors:** Valentin Y Skryabin, Michael Zastrozhin, Dmitry A Sychev

**Affiliations:** 1 Department No. 2, Moscow Research and Practical Centre on Addictions, Moscow, RUS; 2 Laboratory of Genetics and Fundamental Studies, Moscow Research and Practical Centre on Addictions, Moscow, RUS; 3 Clinical Pharmacology and Therapy Department, Russian Medical Academy of Continuous Professional Education of the Ministry of Health of the Russian Federation, Moscow, RUS

**Keywords:** pharmacogenetics, olanzapine, creatine kinase, rhabdomyolysis, personalized medicine, pgx2

## Abstract

This paper presents the case of a 20-year-old patient with a suspected diagnosis of paranoid schizophrenia. He was prescribed oral olanzapine at a dose of 10 mg per day, and the treatment was associated with rhabdomyolysis (serum creatine kinase = 9,725 U/L on day four of the therapy). On suspicion of its contribution to rhabdomyolysis, olanzapine was immediately withdrawn. Pharmacogenetic testing demonstrated that the patient’s *CYP2D6* genotype was *4/*4 (1846G>A, rs3892097). Based on these results, the patient was switched to trifluoperazine, a medication that is not metabolized by the CYP2D6 isoenzyme. Subsequently, the patient recovered well and was discharged without any nephrological sequelae. The presented case demonstrates that pharmacogenetic‐guided personalization of treatment may allow selecting the best medication and determining the right dosage, resulting in the reduced risk of adverse drug reactions and pharmacoresistance.

## Introduction

Today psychiatrists have a greater choice of treatment for schizophrenia than ever. The number of medications has increased substantially over recent years with the development of novel atypical antipsychotics targeting different receptor subtypes. Olanzapine (sold under the trade name Zyprexa) is an antipsychotic initially approved in 1996 by the US Food and Drug Administration (FDA) for the treatment of schizophrenia [1]. This medication is extensively prescribed and is considered to be one of the most efficacious antipsychotics for schizophrenia marketed in the United States and elsewhere [2].

Olanzapine is metabolized primarily by N-glucuronidation mediated by uridine diphosphate glucuronyltransferase [3]. Apart from glucuronidation, olanzapine undergoes oxidative hepatic metabolism resulting in the formation of 4'-N-desmethyl-olanzapine, olanzapine N-oxide, and 2-hydroxy-olanzapine. The formation of 2-hydroxymethyl-OLA is attributed to the CYP2D6 isoenzyme [4]. CYP2D6 activity is genetically regulated, and multifold, interindividual differences in its activity are associated with variant alleles that result in either abolished (e.g., CYP2D6*3, *4, *5, *6, *7, and *8), decreased (e.g., CYP2D6*9, *10, *17, and *41), normal (e.g., CYP2D6*1 and *2), or increased (multiple copies of CYP2D6*1 or *2) enzymatic activity [5]. Accordingly, individuals can be classified as poor metabolizers (PM), intermediate metabolizers, extensive metabolizers (EM), or ultrarapid metabolizers, respectively, based on their genotype. The impaired metabolic capacity of the PM genotype results in higher steady‐state plasma concentrations at a given dose, thus increasing the risk of adverse drug reactions (ADRs) from medication [6].

Rhabdomyolysis is a potential complication of psychotropic drug use and may potentially lead to life-threatening complications, such as acute renal failure [7]. Although rhabdomyolysis is a rare side effect (<1%) of olanzapine, numerous reports showing evidence of the association between regular dosage of olanzapine and rhabdomyolysis have been published to date [8-10]. Furthermore, olanzapine-induced rhabdomyolysis is mentioned in the summary of product characteristics [11].

Rhabdomyolysis is a clinical entity characterized by the destruction of skeletal muscle with a resultant release of intracellular enzymatic content into the bloodstream that leads to systemic complications. The clinical presentation may vary, ranging from an asymptomatic increase in serum levels of enzymes released from damaged muscles to conditions such as volume depletion, metabolic and electrolyte abnormalities, and acute kidney injury (AKI). The diagnosis is confirmed when the serum creatine kinase (CK) level is >1000 U/L or at least five times the upper limit of normal [12].

This paper intends to illustrate the occurrence of olanzapine-associated rhabdomyolysis with a clinical case, as well as to underline the diagnostic approach to elevated CK activity and the potential role of pharmacogenetic testing to predict the ADRs. All steps have been taken to ensure that any identifying details of the patient have been removed.

## Case presentation

A 20-year-old male Caucasian patient was admitted to the Psychiatric Department with a one-month history of irrational behavior, talking to himself, persecutory delusions, and poor sleep. His parents reported that the patient’s behavior had changed during the last few months to a hypervigilant recluse. The patient’s heredity was not burdened by mental disorders or substance abuse, and there was no history of psychoactive substance use. His cognitive state was normal, but the patient had no insight into being mentally ill. A physical examination revealed no particular symptoms, and the neurological state was unremarkable. After a thorough examination, paranoid schizophrenia was suspected (F20.0, according to International Classification of Diseases, Tenth Revision) and the patient was prescribed oral olanzapine at a dose of 10 mg per day. On admission, routine blood tests showed serum CK within the normal range (94 U/L), and serum chemistry and blood count were also within the normal values.

After two days of olanzapine monotherapy, the patient experienced muscle jerks in the legs. Four days after the initiation of olanzapine treatment, he complained about fatigue and weakness in the lower extremities along with myalgia. Physical examination revealed decreased muscle power with no extrapyramidal symptoms. Blood chemistry showed that serum CK and serum lactate dehydrogenase (LDH) levels were markedly elevated (9,725 U/L and 843 U/L, respectively). However, the patient’s body temperature, electrocardiogram, MRI of the brain, complete blood count, troponin T, electrolytes, C-reactive protein, blood urea nitrogen, creatinine, and glucose levels were normal. It allowed excluding other possible causes of an increased CK level, such as myocardial infarction or stroke. The patient had no signs of serotonin syndrome or neuroleptic malignant syndrome. The Naranjo algorithm score of 6 suggested that olanzapine was the probable cause of rhabdomyolysis. Other medications were excluded as probable causes of rhabdomyolysis due to their negative Naranjo algorithm scores. A diagnosis of drug-induced rhabdomyolysis was established from the background of blood tests (increased serum CK and LDH levels), clinical presentation (fatigue and weakness in the lower extremities, muscle jerks, and myalgia), and Naranjo algorithm score of 6 for olanzapine. On suspicion of its contribution to rhabdomyolysis, olanzapine was immediately withdrawn. The patient was referred to the intensive care unit. To prevent acute renal failure, high-volume alkaline diuresis was initiated in accordance with the Russian clinical practice guidelines for the diagnosis and treatment of AKI.

After consulting a clinical pharmacologist, the patient’s primary physician decided to perform a pharmacogenetic test to develop an individualized treatment regimen. Pharmacogenetic test results were interpreted using the PGX2 software (Meditsina LLC, Moscow, Russia). The test revealed that the patient was a homozygous mutant for CYP2D6*4, which corresponds to CYP2D6 PM phenotype. With this in mind, trifluoperazine was prescribed at a daily dose of 10 mg instead of olanzapine as recent data indicate that trifluoperazine is metabolized by CYP1A2 and UGT1A4 instead of CYP2D6.

One month after the switch to trifluoperazine, there was no recurrence of rhabdomyolysis, and clinical and laboratory findings were normal. The patient’s appearance, mood, behavior, perception, and insight improved while on trifluoperazine therapy. The patient was discharged without any nephrological sequelae (serum CK and LDH levels were 205 U/L and 180 U/L, respectively). We suggest that after antipsychotic-induced rhabdomyolysis, a switch to another atypical antipsychotic can be a cautious clinical strategy, and pharmacogenetic testing is extremely important in such patients.

## Discussion

Recent research demonstrates that CYP2D6 is one of the most important isoenzymes implicated in drug metabolism because the *CYP2D6 *gene is highly polymorphic [13]. Approximately 7% to 10% of the European population are CYP2D6 PMs who only weakly metabolize drugs like antiarrhythmics, antidepressants, antipsychotics, and some β-blockers [14]. Among this subgroup, clinically relevant ADRs are more likely compared with EM. Individuals who express poor or a complete lack of enzyme function are predisposed to the accumulation of the parent drug and achieve excessive serum levels and prolonged half-lives of the drugs [15]. These individuals have a tendency to experience ADRs on the “usual” doses of medications. Because ADRs of many antipsychotics are dose-dependent, genotyping may be valuable for patients taking drugs that are primarily metabolized by CYP2D6.

Although unclear, the mechanism of olanzapine-associated rhabdomyolysis might be drug-related myopathy [11]. The underlying mechanism seems to involve the calcium-dependent potassium efflux, which is responsible for membrane hyperpolarization and muscle refractoriness. Olanzapine has a high affinity for histamine-H1, 5-hydroxytryptamine 2A (5-HT2A), and dopamine D2. H1-receptor antihistamines, acting on the sarcolemma, may facilitate sodium flux into the cells, thereby depleting intracellular adenosine triphosphate (ATP) through the activation of energy-dependent Na+/K+ ATPase. Moreover, an increased intracellular sodium concentration can elevate calcium and activate intracellular proteolytic enzymes, causing progressive rhabdomyolysis-related injury to muscle cells [16]. Another postulation concerns the role of serotonin, which accumulates in skeletal muscle by passive diffusion. Tricyclic antipsychotics, including olanzapine, might interact with endogenous 5-HT to cause skeletal muscle injury. The antagonist activity at the 5-HT2A receptors can block the uptake of glucose by skeletal muscles and increase its permeability to CK [17].

Our case report describes how the patient developed rhabdomyolysis after the initiation of therapy with olanzapine. The temporal relationship between olanzapine administration and the occurrence of rhabdomyolysis, with regression of symptoms after the discontinuation of the medication, supports the hypothesis that rhabdomyolysis was caused by olanzapine. Few reports on the association between olanzapine use and rhabdomyolysis have been published to date, and the present case report draws attention to pharmacogenetic testing which allowed the psychiatrist to prescribe another antipsychotic with no risk of rhabdomyolysis. If this had been performed prior to the antipsychotic prescription, the psychiatrist could have avoided the ADRs as the early adaptation of a therapy regimen to genetic traits helps in avoiding ADRs and improve the clinical outcome of pharmacotherapy [14]. In our case, test results were interpreted using PGX2, a bioinformatics cloud service for data analysis and interpretation of a pharmacogenetic test with recommendations understandable to the physician. PGX2 has already shown its relevance in clinical practice [18]. The test demonstrated that the patient’s CYP2D6 genotype is *4/*4 (1846G>A, rs3892097), and the PGX2 report obtained for this patient having CYP2D6 PM phenotype allowed predicting the efficacy and safety of all antipsychotics metabolized in the liver.

This clinical case demonstrates that genetic counselors and/or clinical pharmacologists can play an important role in delivering pharmacogenetic testing by assisting physicians in the use and interpretation of pharmacogenetic information. Scientific sources suggest that a partnership between genetic counselors, clinical pharmacologists, and treating physicians enables the provision of comprehensive pharmacogenetic services, providing information about the appropriate use of testing based on current evidence [19].

## Conclusions

This case report suggests that olanzapine may cause rhabdomyolysis in a proportion of patients who have a pharmacogenetic predisposition. Clinical presentation, increased serum CK and LDH levels, and increased Naranjo algorithm scores can allow the attending physician to establish the diagnosis. Olanzapine withdrawal relieves symptoms and reduces serum CK and LDH levels to normal ranges. Pharmacogenetic‐guided personalization of treatment allows selecting the best medication and determining the right dosage, resulting in the reduced risk of ADRs and pharmacoresistance.

There are some limitations to this study: (1) As it is a single case report, there are no epidemiological data. Thus, we cannot draw any conclusions on the incidence or prevalence levels of the phenomenon described. (2) Findings obtained from the case report cannot be generalized.

The description of such clinical cases demonstrating the cooperation between the clinicians and clinical pharmacologists expands our knowledge in this area, leading to more effective and safe treatment interventions.
